# The Use of Differential Scanning Calorimetry to Study the Thermal Effects of Structurally Similar Pregnane Analogs

**DOI:** 10.1002/cmdc.70563

**Published:** 2026-11-11

**Authors:** Errikos Petsas, Filippos Panteleimon Chatzipieris, Christos T. Chasapis, Thomas Mavromoustakos

**Affiliations:** ^1^ Laboratory of Organic Chemistry Department of Chemistry National and Kapodistrian University of Athens Athens Greece

**Keywords:** differential scanning calorimetry, dipalmitoyl phosphatidylcholine, lipid bilayers, steroids

## Abstract

Differential scanning calorimetry (DSC) is a valuable technique to study drug: membrane interactions. We have studied the thermotropic properties of nine pregnane analogs with known biological activity. Structure activity relationships were revealed for the nine molecules possessing different activities between the lowering of main phase transition (*T*_c_) of dipalmitoyl phosphatidylcholine bilayers and increase of the half width of the main phase transition (Δ*T*_C1/2_). The lowering of *T*_c_ and increase of Δ*T*_c1/2_ broadly paralleled the reported biological activity. This consistent qualitative trend was prominently observed at a molar ratio of 10% (*x* = 0.10), whereas higher steroid concentrations introduced deviations. Thus, it appears that *x* = 0.10 is a compromise concentration to obtain the optimum results. These findings show that DSC is sensitive to subtle stereochemical differences in pregnane analogs, revealing a qualitative correlation with bilayer perturbation, although physical disruption of an idealized DPPC matrix alone does not fully predict in vivo anesthetic potency. More studies are required to reveal the molecular reasons for observing such differences.

## Graphical abstract

Differential scanning calorimetry (DSC) was employed to probe the membrane‐perturbing capacities of nine closely related pregnane analogs incorporated into DPPC bilayers. Main phase transition depressions and peak half‐width broadenings at *x* = 0.10 qualitatively mirror relative in vivo anesthetic potencies, highlighting the utility of DSC as a rapid biophysical tool for evaluating steroid–membrane interactions.

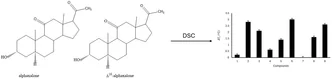

## 1 Introduction

Differential scanning calorimetry (DSC) is an important thermodynamic technique suitable for investigating drug ‐ membrane interactions [1, 2, 3]. Drug: membrane interactions are important in drug action and therefore research activities on this field are related to medicinal chemistry and generally to pharmaceutical research. In DSC experiments, the differential heat flow between a sample of interest and a reference as a function of temperature is measured [4, 5, 6, 7]. This information provides knowledge on the thermally induced phase changes in lipid membranes [8]. In addition, it can also be studied how these phase changes are affected by incorporating pharmacological substances [9, 10, 11, 12].

In our previous study, we had investigated the thermal effects of alphaxalone (5α‐pregn‐3α‐ol‐11,20‐dione) and its structural congener Δ16‐alphaxalone (5α‐pregn‐16‐en‐3α‐ol‐11,20‐dione) using differential scanning calorimetry (DSC) [13].

Our work has been rationalized on the theory that the sites of action of anesthetic steroids are membrane bilayers [14, 15]. In addition, steroids are lipophilic molecules and are expected to embed themselves in the bilayer core [16].

We have shown in our previous work that indeed DSC is a powerful tool to distinguish the potent steroid alphaxalone from the inactive analog Δ^16^‐alphaxalone (Figure 1). The results obtained by DSC were in agreement with other techniques used such as solid‐state NMR spectroscopy and X‐ray diffraction [17, 18].

**FIGURE 1:**
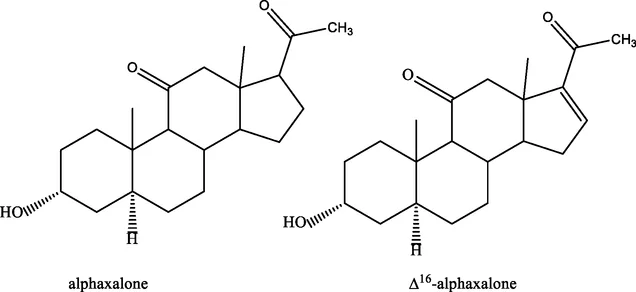
Chemical structure of alphaxalone and Δ^16^‐alphaxalone.

Other authors showed the capability of DSC to differentiate the effects of steroids. For example, the thermal effects of four plant sterols known for their health promoting effects on lowering blood cholesterol levels and alleviating cardiovascular disease were studied. The authors discovered that their thermal changes that caused in the phospholipid model membrane depended mainly on the type of the linker [19] by which stigmatesterol is attached in the glycerol backbone as well as on the type of the hydrocarbon chain.

The thermal effects of cholesterol and five sterol or steroid analogs have been studied [20, 21]. It was found that the characteristic effects of sterols/steroids on fluid–lipid bilayers are optimal when their 3β‐OH group is not oxidized to the carbonyl group. In addition, the presence of a single double bond at C5 is required to maximize sterol solubility in fluid DPPC (dipalmitoylphosphatidylcholine) bilayers, which is probably its primary function in natural sterols.

Moreover, progesterone analogs have been shown to exert distinct effects on DPPC bilayers, indicating that DSC is capable to differentiate these small structural differences [22].

In recent years, complementary biophysical investigations combining differential scanning calorimetry with solid‐state NMR, small‐angle X‐ray scattering, and molecular dynamics simulations have further elucidated the molecular mechanisms governing steroid– and neurosteroid–membrane interactions, detailing how steroid partitioning alters lipid bilayer order, phase transitions, and lateral organization [23]. In parallel, advances in cryo‐electron microscopy and structural pharmacology of the GABA_A_ receptor have identified specific inter‐ and intrasubunit transmembrane binding pockets for neuroactive steroids, emphasizing that membrane bilayer partitioning and local lipid–steroid interactions serve as critical modulators for presenting these molecules to their receptor target sites [24, 25].

This research work is a continuation of our previous work on the two analogs alphaxalone and Δ^16^‐alphaxalone. We have included in our study more molecules which varied in their potency. As the D and B rings were already studied, we focused our attention on the A and C rings. Figure 2 shows the differences of the structures in these two rings.

**FIGURE 2:**
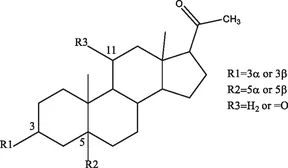
Structures of the studied compounds.

## 2 Materials and Methods

### 2.1 Materials

Alphaxalone and Δ^16^‐alphaxalone were kindly donated by Glaxo Group Research, Middlesex, UK. DPPC was obtained by Avanti Polar Lipids, Birmingham, AL, USA. All other steroids and highly pure chloroform have been purchased by Merck, France.

The calorimeter used was DSC‐7 from Perkin–Elmer (Shelton, CT, USA).

### 2.2 Methods

The lipid with or without steroid was dissolved in chloroform. Steroid content throughout the study is expressed as the steroid mole fraction, defined as *x* equals moles of steroid divided by total moles of lipid plus steroid (corresponding to a 1:9 steroid‐to‐lipid ratio at *x* = 0.10). The solvent is then evaporated by rotavapor under vacuum (0.1 mmHg) at a temperature of 50 °C, which is above the main phase transition of the phospholipid used (1,2‐dipalmitoyl‐sn‐glycero‐3‐phosphocholine (DPPC)) (DPPC *T*_c_ = 41.2 °C). For measurements, this dry residue is dispersed in appropriate amounts of bidistilled water by vortexing above the transition temperature to form fully hydrated multilamellar bilayers at a 50% w/w water content (excess water condition). An aliquot of the hydrated dispersion (ca. 5.0 mg total sample mass, containing approximately 2.5 mg dry DPPC) in order to form fully hydrated bilayers (50% w/w). An aliquot of the hydrated sample (ca. 5 mg) was hermetically sealed into stainless‐steel capsules (60 μL capacity, PerkinElmer, Norwalk, CT, USA) equipped with gold‐plated copper seals to prevent water loss during scanning.

Thermal scans were recorded over the temperature range of 15–50 °C. Before scanning, the samples were held isothermally at 50 °C for 2 min to ensure complete thermal equilibration in the fluid liquid‐crystalline (*L*_α_) phase and to eliminate any prior thermal history, thereby promoting a uniform distribution of the steroid within the DPPC matrix. Each capsule underwent consecutive heating and cooling scans at a constant scanning rate of 2.5 °C/min. Samples were routinely cycled until identical, superimposable thermograms were obtained (typically within 3–5 consecutive runs), confirming thermal reproducibility and verifying the absence of sample degradation or moisture leakage. Cooling scans run at the same rate exhibited the expected thermodynamic hysteresis without divergent phase behavior; therefore, consecutive heating scans were utilized for baseline correction and the extraction of calorimetric parameters. The scan rate of 2.5 °C/min was selected as an optimal compromise providing well‐resolved, undistorted peak profiles and a high signal‐to‐noise ratio while minimizing kinetic peak broadening and instrumental thermal lag, consistent with previously established protocols for lipid‐steroid assemblies. All experiments were carried out using two completely independent sample preparations (independent duplicates, *n* = 2) prepared from separate lipid–steroid stock mixtures and hydration procedures. Each sealed capsule was scanned at least twice consecutively at a scanning rate of 2.5 °C/min to confirm thermal reproducibility and eliminate prior thermal history. Calorimetric parameters were extracted from the recorded thermograms using the PerkinElmer DSC software. The main phase transition temperature (*T*_c_) was defined as the peak maximum of the main endothermic event. The transition half‐width (Δ*T*_c1/2_) was evaluated as the full peak width at half‐maximal peak height (FWHM). For thermograms exhibiting asymmetric profiles, broad shoulders, or overlapping multicomponent features (notably observed for compound 3), *T*_c_ was systematically recorded at the primary heat capacity maximum, and Δ*T*_c1/2_ was evaluated across the total apparent transition envelope at half the maximum amplitude of the predominant endotherm, avoiding ambiguous single‐peak deconvolution.

Transition enthalpy (Δ*H*) was calculated by numerical integration of the transition peak area against a linear baseline drawn between the pre‐ and posttransition regions, calibrated against high‐purity standard indium. Transition temperatures and transition half‐widths demonstrated an experimental difference of 0.20 °C between runs, corresponding to a sample standard deviation of ±0.14 °C across independent duplicate preparations (*n* = 2). Enthalpy determinations yielded an experimental variability of ±0.42–0.57 cal/g (±1.3–1.7 kJ/mol). Individual replicate values for all parameters are reported in Table S1.

## 3 Results and Discussion

We will provide comparative DSC studies for all molecules studied. We will separate these analogs in groups in order to make the comparisons easier. Table 1 are shown the anesthetic activities of the compounds studied.

**TABLE 1:** Pregnane analogs: The structures and anesthetic activities of the pregnane steroids.

Steroid	*R*_1_	*R*_2_	*R*_3_	16,17 double bond	Anesthetic ED, mg/kg^a^
1.	3*β*OΗ	5*β*Η	Η_2_	No	25
2.	3*α*OΗ	5*β*Η	Η_2_	No	3.1
3.	3*α*OΗ	5*α*Η	Η_2_	No	3.1
4.	3*β*OΗ	5*α*Η	Η_2_	No	100
5.	3*α*OΗ	5*β*H	═O	No	6.3
6.	3*α*OΗ	5*α*Η	═O	No	3.1
7.	3*α*OΗ	5*α*Η	═O	Yes	Inactive^b^
8.	3*β*OΗ	5*α*Η	Η_2_	Yes	Inactive^c^
9.	3*β*OΗ	5*α*Η	═O	Yes	Inactive^c^

a Minimum effective dose (ED, mg/kg) producing loss of righting reflex in mice following intravenous (i.v.) administration.

b Data taken from Phillips (1975) [26].

c Data taken from Davis (1975) [27].

First, comparing the two pregnane analogs 3*β*‐hydroxy‐5*β*‐pregnan‐20‐one and 3*α*‐hydroxy‐5*α*‐pregnan‐20‐one (Figure 3), this pair of analogs differs only in the orientation of the 3OH group and the 5‐H atom at the A/B ring junction. This structural difference makes 3*α*‐hydroxy‐5*α*‐pregnan‐20‐one (compound **3** in Table 1) much more active than 3*β*‐hydroxy‐5*β*‐pregnan‐20‐one (compound **1** in Table 1).

**FIGURE 3:**
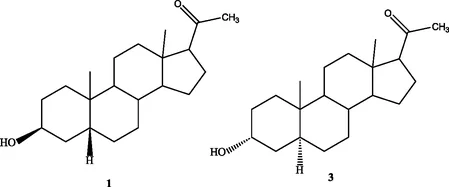
Pregnane analogs: Chemical structures of two epimeric pregnane analogs 3β‐hydroxy‐5β‐pregnan‐20‐one (**1**) and 3α‐hydroxy‐5α‐pregnan‐20‐one (**3**).

The thermal effects of DPPC bilayers alone and in combination with each of the two studied compounds **1** and **3** are presented in Figure 4.

**FIGURE 4:**
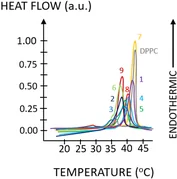
Normalized baseline‐corrected DSC thermograms of DPPC bilayers containing pregnane analogs **1–9** at *x* = 0.10 overlaid with pure DPPC (gray) recorded at a scanning rate of 2.5 °C/min. Curves are normalized to maximum peak intensity (in arbitrary units) to facilitate visual comparison of phase transition profiles and peak broadening across all studied steroids. Thermograms are color‐coded for clarity: compound **1** (purple), compound **2** (dark blue), compound **3** (blue), compound **4** (cyan), compound **5** (green), compound **6** (light green), compound **7** (orange), compound **8** (red), compound **9** (deep red), and pure DPPC (gray).

The first thermogram shows the thermal profile of DPPC bilayers. Thermogram contains a broad peak at *ca* 36.9 °C, which is called the pretransition prior to the main of transition. A sharper peak presenting the main phase transition appears at ca 41 °C. Below the pretransition, phospholipid molecules are arranged in a one‐dimensional lamellar gel phase (*L*_β_)'. Above the main phase transition, they exist in the liquid‐crystalline phase (*L*_α_). At temperatures between *T*_pre_ and *T*_c_ (*T*_pre _< *T*_c_), there is a ripple phase (*P*_β'_) which, on the basis of solid‐state nuclear magnetic resonance (NMR) evidence, has been shown to be made up of gel and liquid‐crystalline components [28].

The presence of *x* = 0.10 (molar ratio 10%) of 3β‐hydroxy‐5β‐pregnan‐20‐one (compound **1** in Tables 1 and 2) causes a slight lowering of *T*_c_ (main‐phase transition) by 0.2 °C, an increase of Δ*T*_c1/2_ (half‐width of the peak) by 1.1 °C, and an increase in Δ*Η* by 0.4 cal/g (1.2 kJ/mol). The pretransition is abolished, indicating that incorporation of the steroid disrupts the periodic ripple phase (*P*_B’_) by localizing near the polar/apolar interfacial boundary of the phospholipid headgroups. The presence of the same molar ratio (10%) of the more active analog 3α‐hydroxy‐5α‐pregnan‐20‐one (compound **3** in Table 1) exerts much more significant effects. It shifts *T*_c_ downward by 2.1 °C, broadens Δ*T*_c1/2_ by 2.8 °C, and likewise eliminates the pretransition. Interestingly, Δ*Η* remains virtually unaffected compared to DPPC bilayers alone (a negligible change of only 0.1 cal/g or 0.3 kJ/mol) (Table 2). This minimal enthalpy change indicates that although compound **3** substantially reduces the cooperativity of the phase transition, it does not impede the overall energetic cost of trans‐to‐gauche conformational isomerizations within the hydrophobic core.

**TABLE 2:** Calorimetric data for the pregnane analogs used at *x* = 0.1: melting temperature *T*_c_, half‐width Δ*T*_c1/2_, and enthalpy change Δ*Η* for DPPC/steroid (*x* = 0.10).

Compound	***T***_**c**_ **, °C**	**Δ*T***_**c1/2**_ **, °C**	Δ*H*, cal/g	Δ*H*, kJ/mol
**1**	41.0	2.3	10.5	32.2
**2**	38.4	2.8	10.4	31.9
**3**	39.1	4.0	10.2	31.3
**4**	40.6	1.9	9.0	27.6
**5**	39.8	2.8	10.3	31.6
**6**	38.2	2.8	11.7	35.9
**7**	42.0	1.6	11.9	36.5
**8**	39.6	1.6	8.4	25.8
**9**	38.6	1.6	8.9	27.3
**DPPC**	41.2	1.2	10.1	31.0

From the comparison of this pair, it is evident that these epimeric compounds exert differential thermal effects on the lipid membrane. Thermogram C displays not only a markedly broadened transition but also an asymmetric peak profile with a discernible shoulder. Rather than demonstrating a definitive mechanism, this multicomponent footprint can tentatively be framed as a working hypothesis: it may reflect lateral phase heterogeneity, potentially arising from steroid‐rich and steroid‐poor lipid domains, or multiple intercalation modes within the bilayer interface. However, dedicated complementary biophysical investigations (such as solid‐state NMR or X‐ray diffraction) are required to experimentally substantiate lateral domain coexistence or distinct binding topographies.

To further investigate the role of the double bond at position sixteen, we compared alphaxalone with not only Δ^16^‐alphaxalone but also with two other analogs. Thus, active alphaxalone was compared with the inactive analogs **7–9**. Figure 5 shows the structures of the four compounds. As it can be seen, the thermal profile of compound **6** (alphaxalone) resembles that of compound **3** (Figure 6). It shifts *T*_c_ by 3 °C and increases Δ*T*_c1/2_ by 1.6 °C, compared to DPPC alone. Δ*Η* increases by 1.6 cal/g in comparison with DPPC bilayers alone (Table 2). The inactive analogs **7–9** show pretransitions as DPPC bilayers alone. The Δ*T*_c1/2_ is only broader by 0.4 °C, and *T*_c_ ranges from 38.6 to 42.0 °C (specifically, 42.0 °C for 7, 39.6 °C for 8, and 38.6 °C for 9). Their Δ*H* ranged from 8.9 to 11.9 cal/g.

**FIGURE 5:**
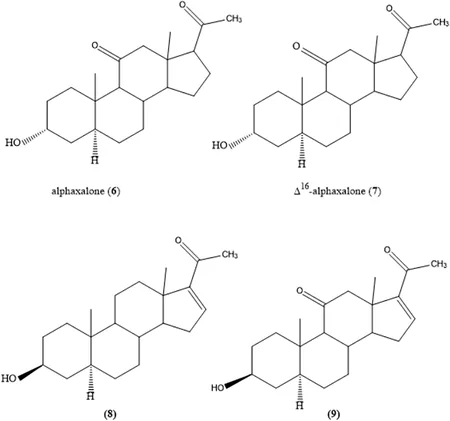
Structures of pregnane analogs **6–9**.

**FIGURE 6:**
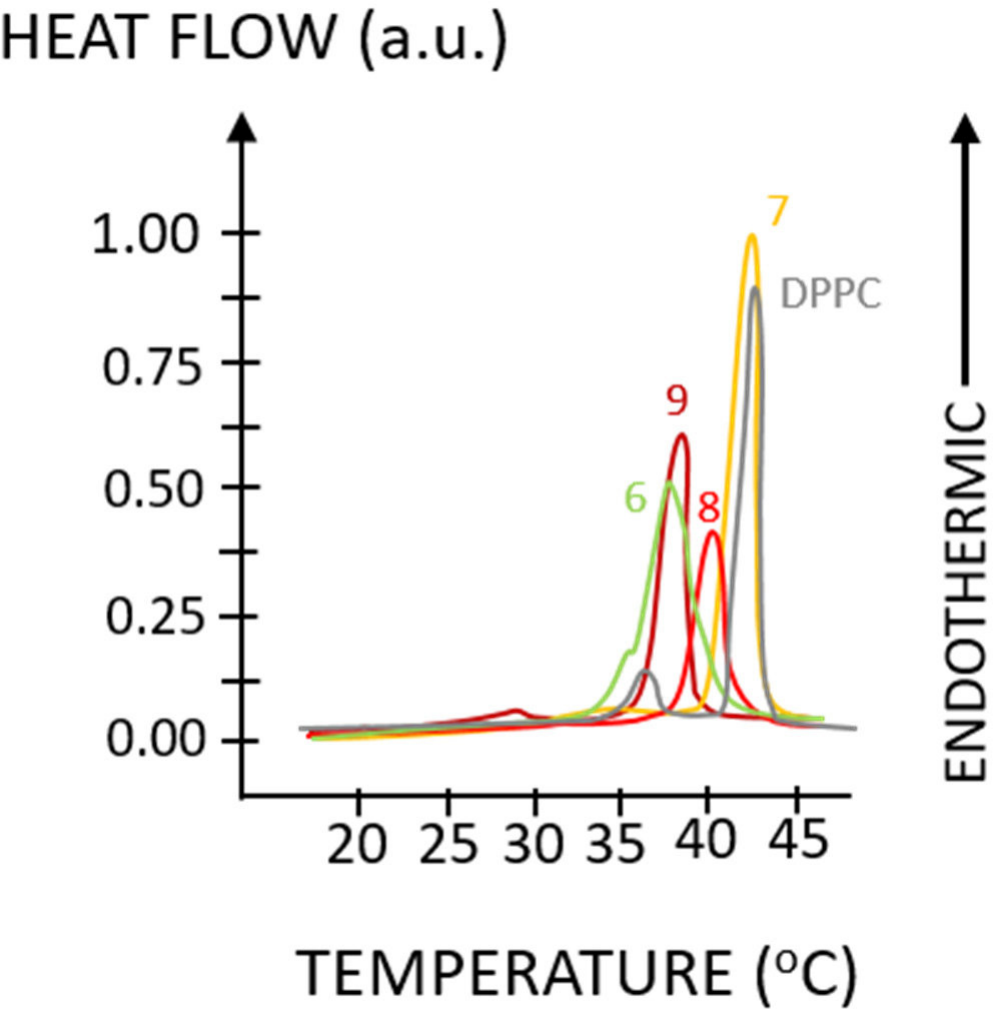
DPPC bilayers containing *x* = 0.1 of either one of the compounds **6–9**. The thermal scans were obtained on DSC‐7 Perkin Elmer with a scanning rate of 2.5 °C/min. Thermograms for all compounds are depicted in different color for clarity (Compound **6**: light green, Compound **7**: orange, Compound **8**: red, Compound **9**: deep red, DPPC: gray).

Values represent mean values from independent duplicate preparations (*n* = 2) with sample standard deviations of ±0.14 °C for *T*_c_ and Δ*T*_c1/2_. Detailed individual replicate data for all calorimetric parameters are reported in Table S1. Enthalpy values in kJ/mol were calculated using 1 cal = 4.184 J and Mw(DPPC) = 734.04 g/mol.

We have compared the thermal effects of **2, 4,** and **5** (Figure 7).

**FIGURE 7:**
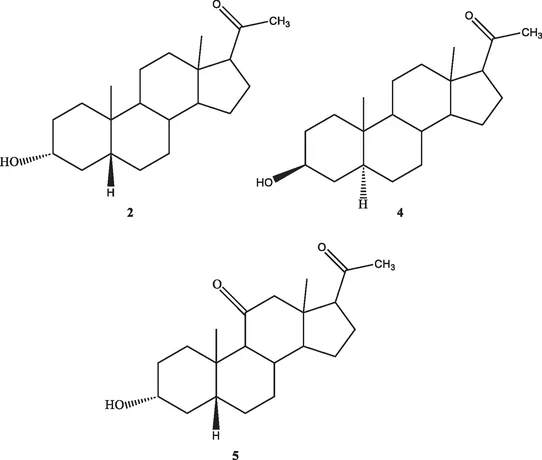
Structures of pregnane analogs **2, 4,** and **5**.

Again, the same trend has been observed. The highest in activity **2** showed the most decrease of *T*_c,_ while the least active compound **4**, showcased the smallest decrease of *T*_c_. Compound **5** showed an intermediate decrease (see Figure 8). The same applies also for the increase of Δ*T*_c1/2_.

**FIGURE 8:**
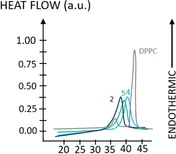
DPPC bilayers containing *x* = 0.1 of either one of the compounds **2, 4,** and **5**. The thermal scans were obtained on DSC‐7 Perkin Elmer with a scanning rate of 2.5 °C/min. Thermograms for all compounds are depicted in different color for clarity (Compound 2: deep blue, Compound 4: light blue, Compound 5: green, DPPC: gray)**.**

To quantitatively evaluate the relationship between membrane perturbation and biological activity, a Spearman rank‐order correlation analysis was conducted for the nine pregnane analogs at *x* = 0.10. Analogs were ranked according to their reported anesthetic potency (compounds **2**, **3**, and **6** sharing the highest tied rank, followed by compound **5**, weak compounds **1** and **4**, and inactive compounds **7–9** assigned tied ranks in the lowest tier). The correlation between anesthetic potency and transition peak broadening (Δ(Δ*T*_c1/2_)) proved statistically significant (rs = 0.77, *p* = 0.015), corroborating that loss of cooperativity in DPPC bilayers broadly parallels anesthetic efficacy, with all three inactive congeners (**7–9**) exhibiting the smallest broadening (Δ(Δ*T*_c1/2_) = 0.4 °C). In contrast, the correlation with the main transition temperature depression (Δ*T*_c_) was weaker and did not reach statistical significance (rs = 0.61, *p* = 0.082). This divergence stems from notable exceptions within the series: inactive analogs **8** and **9** induce substantial *T*c depressions (Δ*T*_c _= 1.6 and 2.6 °C, respectively), which exceed that of intermediate analog **5** (Δ*T*_c _= 1.4 °C) and weakly active analogs **1** and **4** (Δ*T*_c _= 0.2 and 0.6 °C, respectively), while lacking the pronounced peak broadening observed for active congeners. Furthermore, while compound **5** displays intermediate in vivo potency (ED = 6.3 mg/kg), its transition broadening (Δ(Δ*T*_c1/2_) = 1.6 °C) is identical to that observed for the highly potent analogs 2 and 6. These deviations underscore that while DSC sensitively captures gross stereochemical perturbations of bilayer cooperativity, thermotropic shifts in an idealized single‐lipid model cannot be regarded as an absolute quantitative predictor of in vivo anesthetic activity.

Subsequently, we evaluated the concentration dependance across all nine analogs (**1–9**) at higher molar ratios of *x* = 0.20 and *x* = 0.40 in order to verify whether the thermodynamic trends observed at *x* = 0.10 were maintained at elevated steroid loads. While a clear qualitative SAR trend was evident at *x* = 0.10, increasing the concentration to *x* = 0.20 and *x* = 0.40 revealed notable discrepancies and nonlinear deviations, likely arising from steric saturation or phase separation within the bilayer matrix (Figures S1–S5).

It is important to emphasize that fully hydrated DPPC bilayers serve as an idealized, single‐component model membrane. While native biological membranes display higher complexity due to the presence of varied phospholipid classes, differing acyl chain lengths, and cholesterol, the DPPC matrix provides a highly cooperative, well‐characterized thermodynamic benchmark (*T*_c_ = 41.2 °C). This homogeneity is essential for systematically isolating and resolving the subtle, intrinsic membrane‐perturbing effects arising strictly from stereochemical variations in the pregnane scaffold, free from confounding factors such as lateral phase separation or competing sterol interactions. The clear baseline trends identified here at *x* = 0.10 warrant future investigations into more complex lipid environments, including DPPC/cholesterol mixtures and alternative phospholipid matrices (e.g. DPPE), to elucidate how membrane composition modulates these –steroid–lipid interactions.

## 4 Conclusions

This study explores the qualitative relationship between pregnane stereochemistry and DPPC bilayer perturbation at *x* = 0.10. While the most potent anesthetic steroids (such as **2**, **3,** and **6**) prominently disrupt membrane cooperativity—evidenced by marked transition broadening—the relationship is not strictly monotonic across all analogs.

Specifically, the most active analogs (**2**, **3**, and **6**) produced pronounced depressions in *T*_c_ alongside significant peak broadening. However, notable deviations are observed within the series: inactive analogs 8 and 9 induce substantial downward shifts in *T*c (1.6 and 2.6 °C, respectively), which exceed the shift caused by intermediate analog **5** (1.4 °C) and weakly active analogs **1** and **4** (0.2 and 0.6 °C, respectively). On the other hand, peak broadening (Δ(*Δ* *T*_c1/2_)) correlated significantly with anesthetic potency (rs = 0.77, *p* = 0.015), as all three inactive congeners (**7–9**) showed the smallest broadening (0.4 °C). Intermediate analog **5** (ED = 6.3 mg/kg) caused a moderate depression in *T*_c_ and a peak broadening comparable to the highly active analogs (Figure 9).

The same trend has been observed for the same analogs in increasing Δ*T*_c1/2_. The most active **2, 3, 5, and 6** increased more Δ*T*_c1/2,_ while the inactive ones (**7–9**) showed the least increase. The less active ones **1, 4,** and **5** generally showed intermediate values (Figure 10).

These findings demonstrate that DSC serves as a rapid, cost‐effective, and sensitive biophysical tool capable of probing membrane‐perturbing capacities that correlate well with the pharmacological potency of structurally similar pregnane analogs.

The distinct thermodynamic effects observed among the pregnane analogs at *x* = 0.10 can tentatively be rationalized through working hypotheses based on steroid stereochemistry and packing considerations. For the active epimer 3α‐hydroxy‐5α‐pregnan‐20‐one(3), the planar A/B‐*trans* ring geometry combined with an axial 3α‐hydroxyl group may perturb acyl chain packing, consistent with a marked downward shift in *T*_c_ and extensive peak broadening (Figure 9). However, ring junction geometry alone cannot fully explain membrane behavior, as the bent A/B‐*cis* congener 2 also induces a pronounced lowering of *T*_c_, in contrast to the minor perturbations produced by its 5β congener 1. Furthermore, while the elimination of the DPPC pretransition indicates the disruption of the periodic ripple phase (Pβ), this calorimetric finding alone is insufficient to conclusively assign steroid depth or interfacial localization. Consequently, these mechanistic proposals remain speculative hypotheses that warrant verification by complementary structural and biophysical techniques, such as solid‐state NMR, FTIR spectroscopy, fluorescence anisotropy, or moleculardynamics simulations.

**FIGURE 9:**
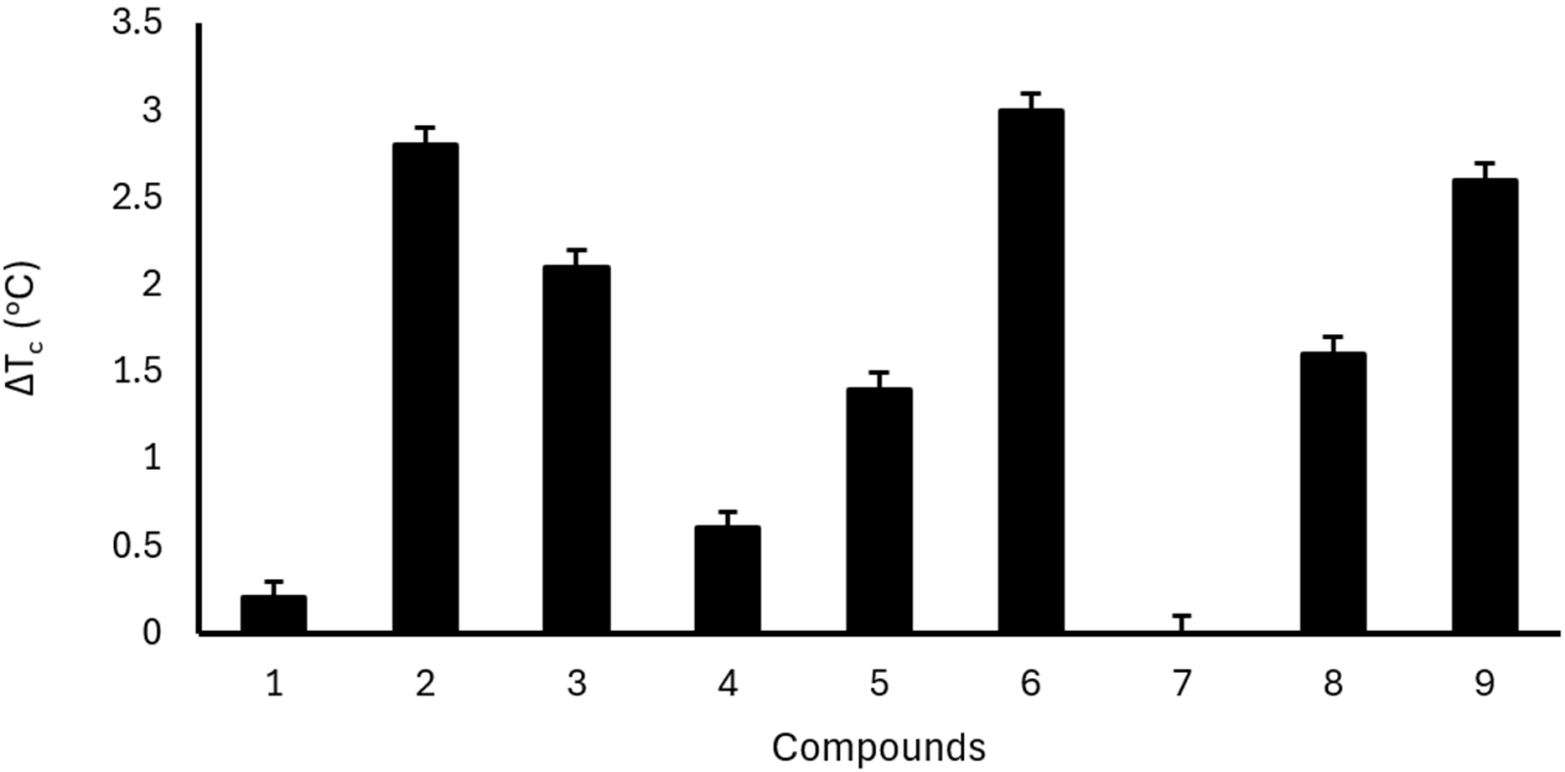
Main phase transition temperature depression (Δ*T*_c _= *T*_c,DPPC_‐*T*_c_) of DPPC bilayers induced by pregnane analogs **1–9** at *x* = 0.10. Values represent the net shift relative to pure DPPC (*T*_c_ = 41.2 °C). Error bars represent standard deviations (SD = ±0.1 °C) from duplicate experiments.

**FIGURE 10:**
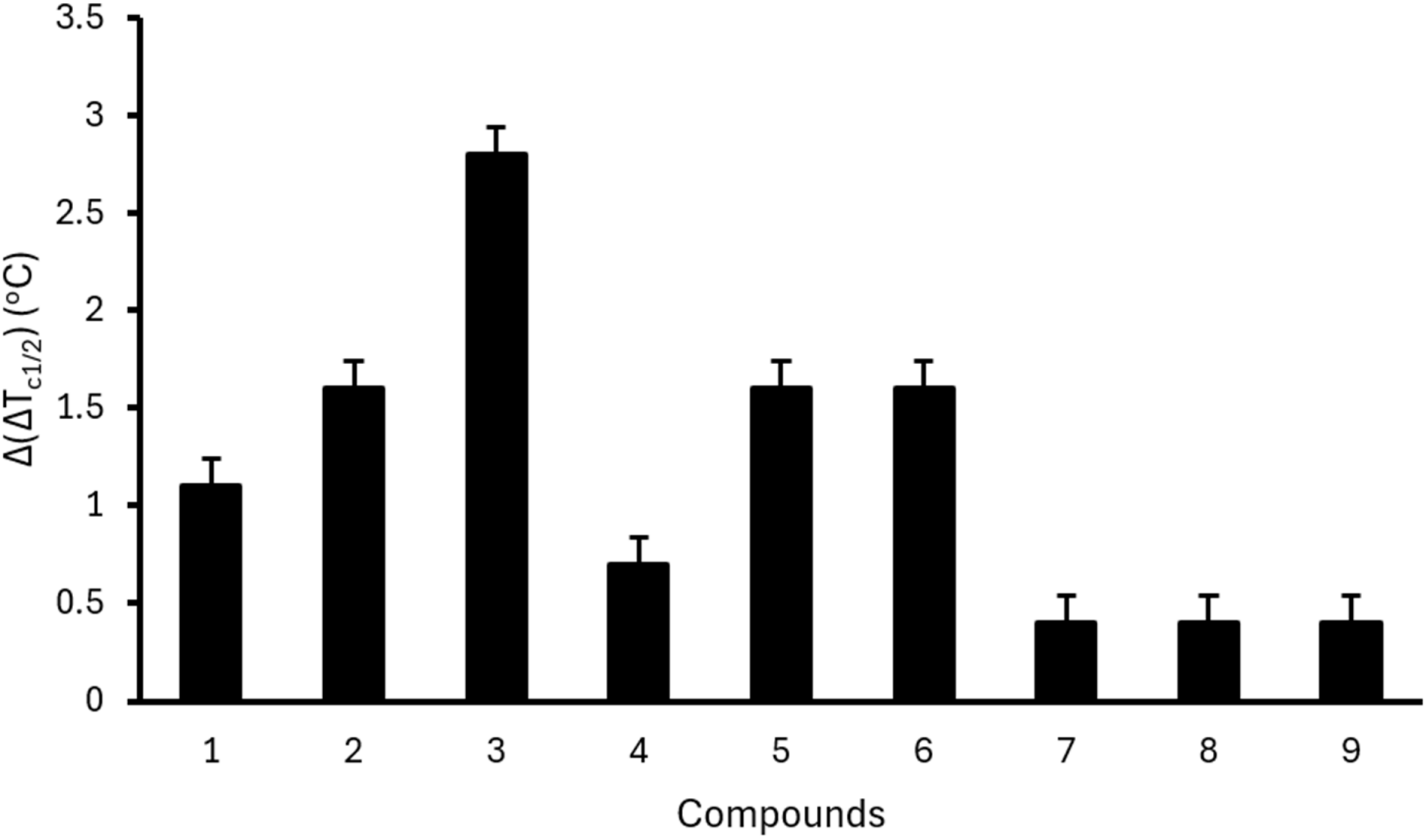
Main phase transition peak broadening (Δ(Δ*T*_c1/2_) = Δ*T*_c1/2_–Δ*T*_c1/2,DPPC_) of DPPC bilayers induced by pregnane analogs **1–9** at *x* = 0.10 shown as a bar chart. Plotted values represent the net increase in half‐width relative to pure DPPC bilayers (Δ*T*_c1/2,DPPC_ = 1.2 °C). Error bars represent sample standard deviations (±0.14 °C) from independent duplicate preparations (individual replicate values and standard deviations are compiled in Table S1).

## Author Contributions

**Errikos Petsas**: methodology, investigation, data curation, writing – original draft. **Filippos Panteleimon Chatzipieris:** methodology, investigation, data curation, writing – original draft. **Christos T. Chasapis**: writing – review and editing. **Thomas Mavromoustakos**: supervision, conceptualization, methodology, investigation, data curation, writing – original draft, writing – review and editing.

## Funding

The authors have nothing to report.

## Conflicts of Interest

The authors declare no conflicts of interest.

## Supporting information

Supplementary Material

## Data Availability

The data that support the findings of this study are available from the corresponding author upon reasonable request.
