# Supplementary material for: The Use of Differential Scanning Calorimetry to Study the Thermal Effects of Structurally Similar Pregnane Analogs
Source: ChemMedChem. 2026 Nov 11;21(21):e70563. doi: 10.1002/cmdc.70563 (PMC13754535; doi:10.1002/cmdc.70563)
Supplement: Supplementary file 1 [file CMDC-21-e70563-s001.pdf]

# The Use of Differential Scanning Calorimetry to Study the Thermal Effects of Structurally Similar Pregnane Analogs

**Errikos Petsas [1,+], Filippos Panteleimon Chatzipieris [1,+], Christos T. Chasapis [1], Thomas Mavromoustakos [1,*]**

[1]Laboratory of Organic Chemistry, Department of Chemistry, National and Kapodistrian University of Athens, Panepistimiopolis Zografou, 15771 Athens, Greece

[*]Correspondence: tmavrom@chem.uoa.gr

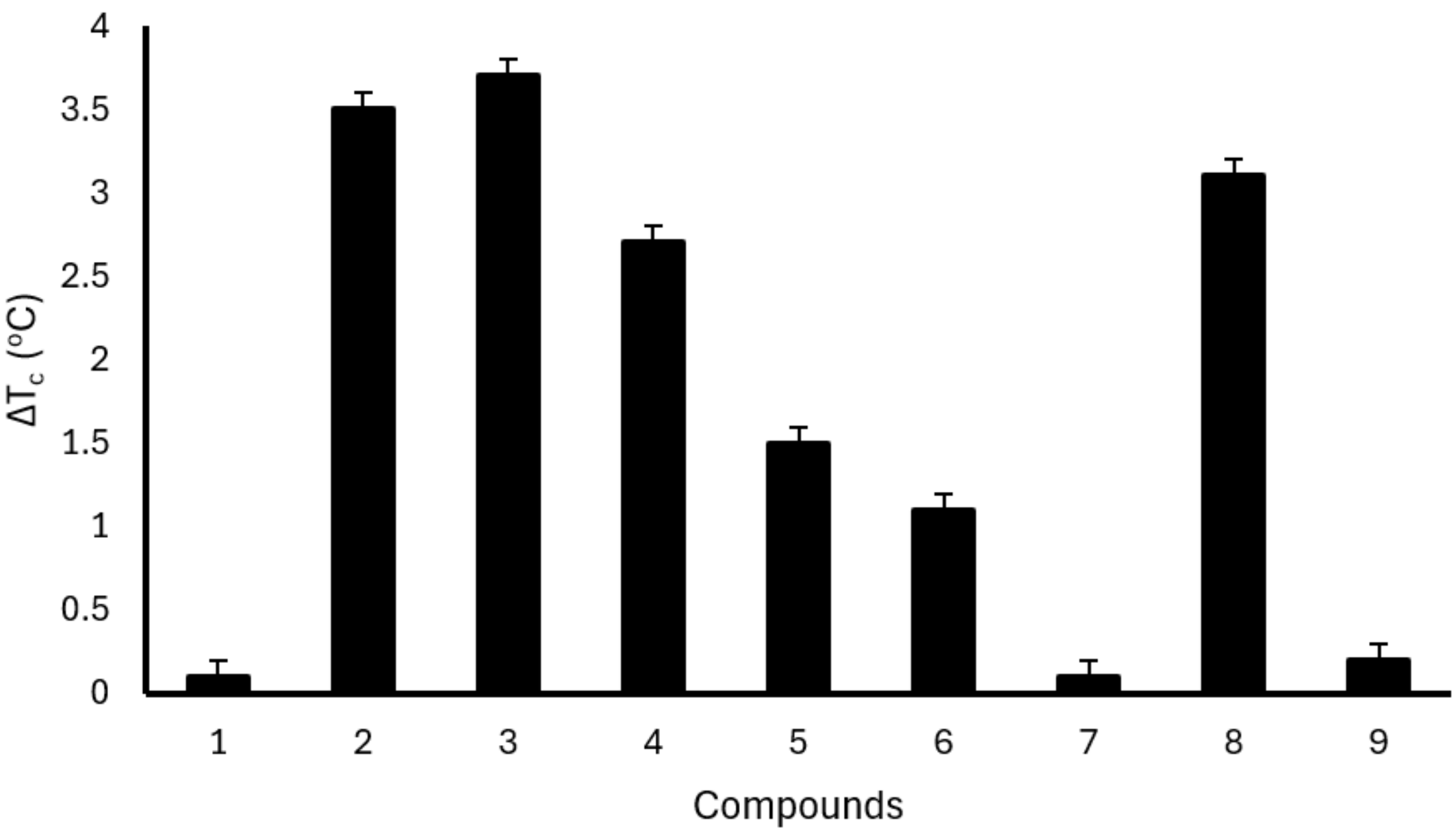


**Figure S1:** Main phase transition temperature depression ($\Delta T_c = T_{c,DPPC} - T_c$) of DPPC bilayers induced by pregnane analogues **1–9** at x = 0.20 shown as a bar chart. Values represent the net shift relative to pure DPPC (Tc = 41.2 °C). Error bars represent sample standard deviations (plus or minus 0.14 °C) from independent duplicate preparations (individual replicate values and standard deviations are compiled in Table S1).

[+]These authors contributed equally to this work.

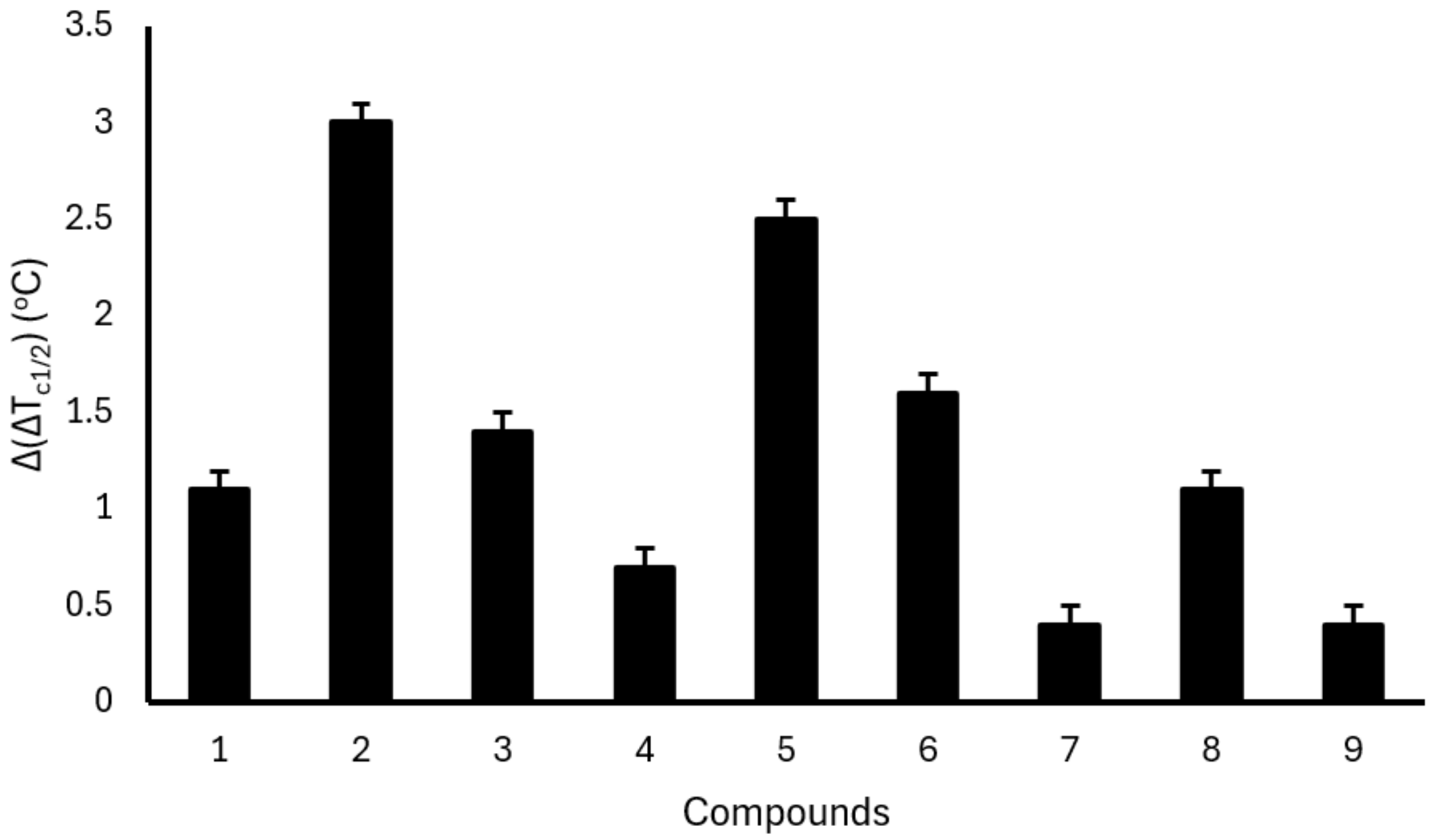


**Figure S2:** Main phase transition peak broadening ($\Delta(\Delta T_{c1/2}) = \Delta T_{c1/2} - \Delta T_{c1/2,\ DPPC}$) of DPPC bilayers induced by pregnane analogues **1–9** at x= 0.20. Plotted values represent the net increase in half-width relative to pure DPPC bilayers $\Delta T_{c1/2}$= 1.2 °C.

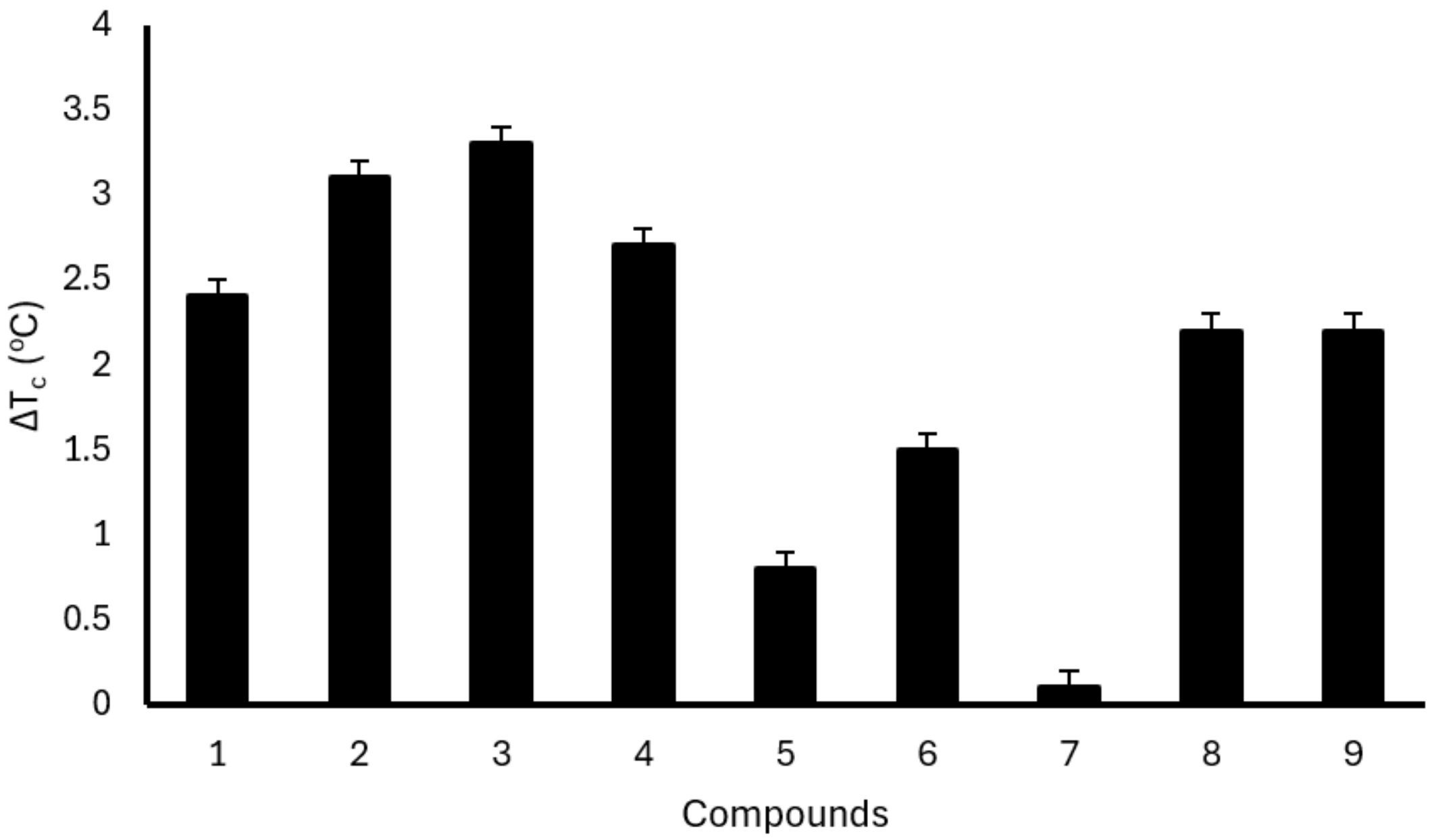


**Figure S3:** Main phase transition temperature depression ($\Delta T_c = T_{c,DPPC} - T_c$) of DPPC bilayers induced by pregnane analogues **1–9** at x = 0.40 shown as a bar chart. Values represent the net shift relative to pure DPPC (Tc = 41.2 °C). Error bars represent sample standard deviations (plus or minus 0.14 °C) from independent duplicate preparations (individual replicate values and standard deviations are compiled in Table S1).

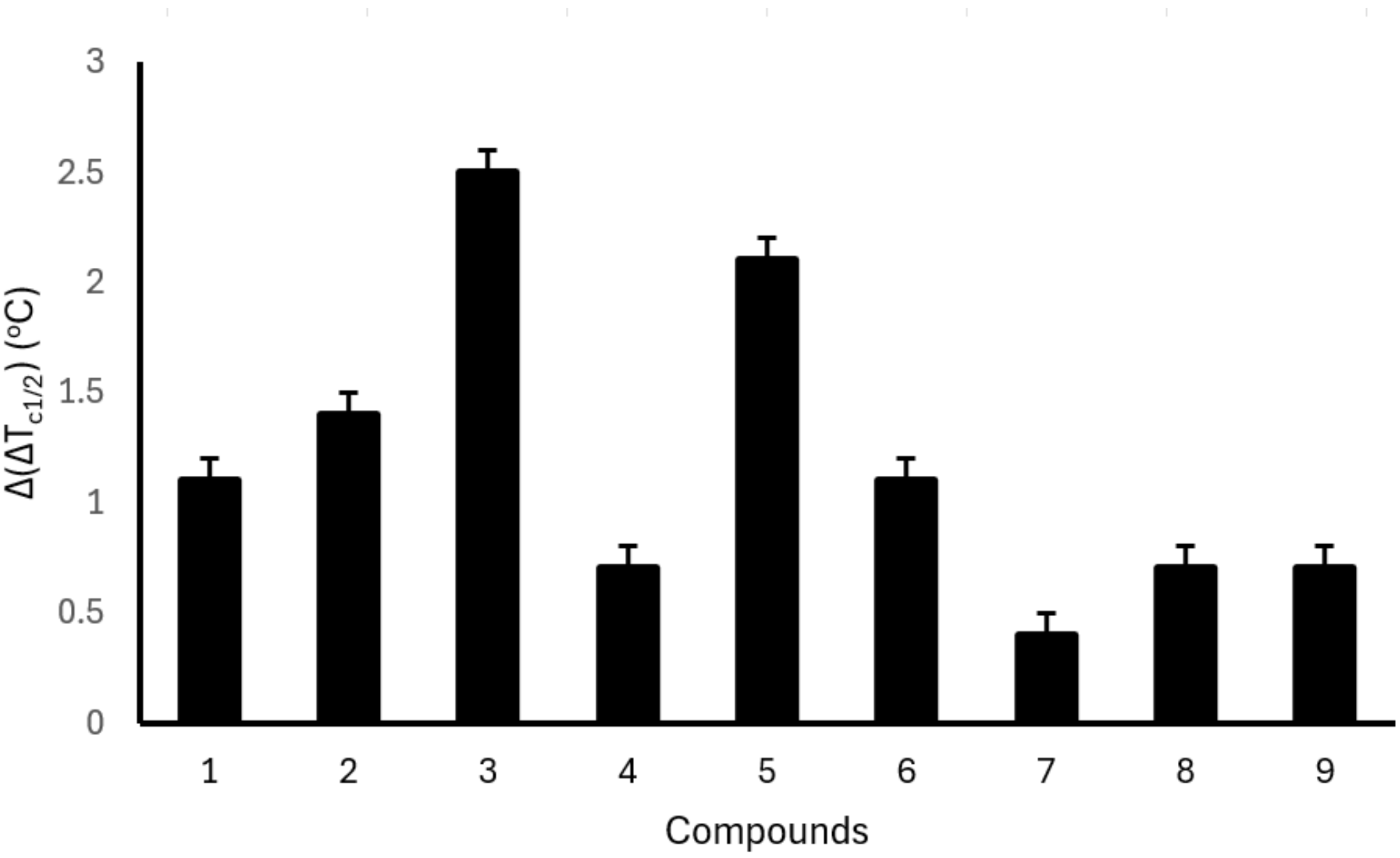


**Figure S4:** Main phase transition peak broadening ($\Delta(\Delta T_{c1/2}) = \Delta T_{c1/2} - \Delta T_{c1/2,DPPC}$) of DPPC bilayers induced by pregnane analogues **1–9** at x = 0.40 shown as a bar chart. Plotted values represent the net increase in half-width relative to pure DPPC bilayers (ΔTc1/2,DPPC = 1.2 °C). Error bars represent sample standard deviations (± 0.14 °C) from independent duplicate preparations (individual replicate values and standard deviations are compiled in Table S1).

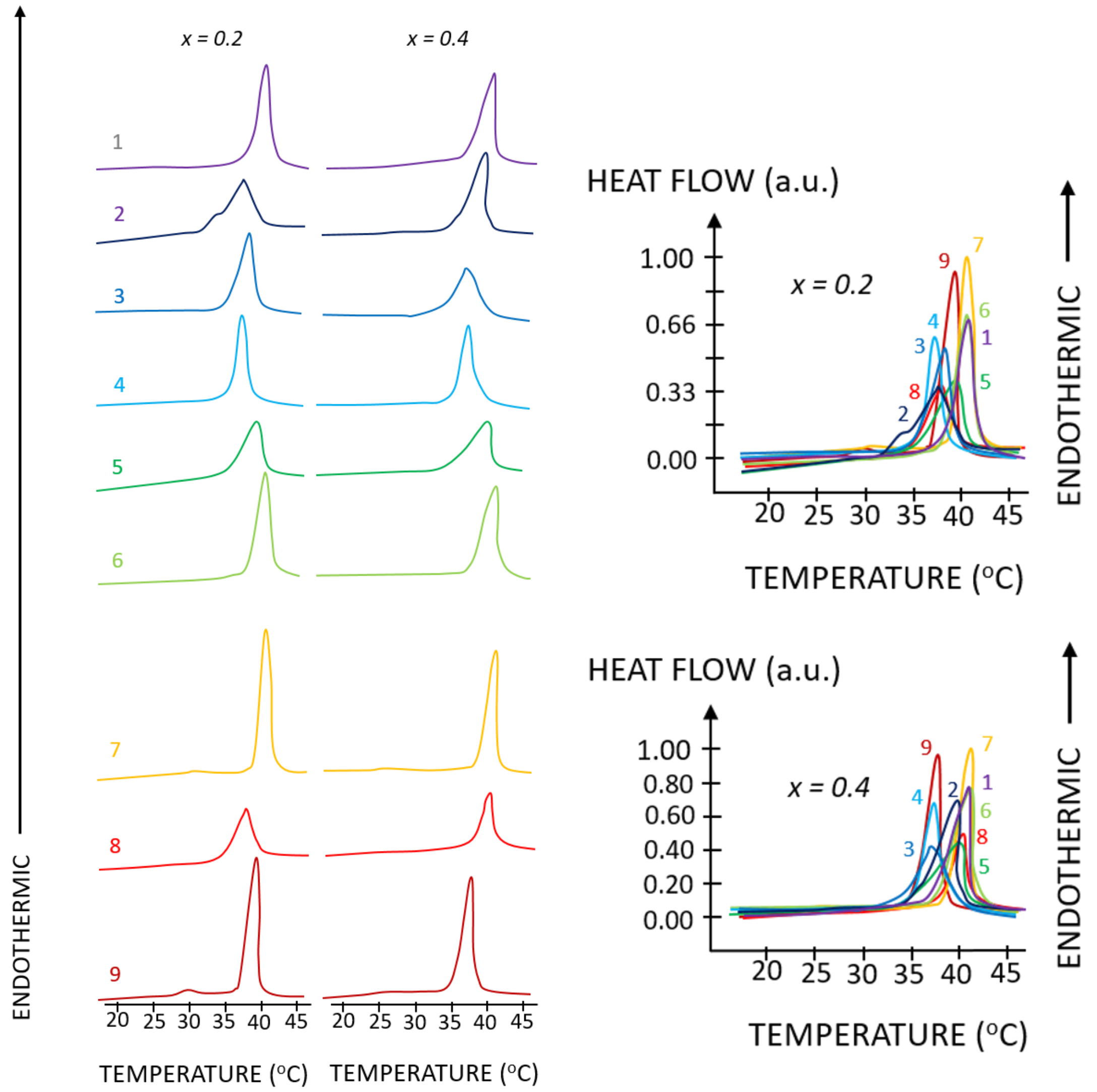


**Figure S5:** DPPC bilayers containing $x$ = 0.2 and $x$ = 0.4 of either one of the compounds **1**-**9**. The thermal scans were obtained on DSC-7 Perkin Elmer with a scanning rate of 2.5 °C/min. Thermograms for all compounds are depicted in different color for clarity (Compound 1: purple, Compound 2: deep blue, Compound 3: blue, Compound 4: light blue, Compound 5: green, Compound 6: light green, Compound 7: orange, Compound 8: red, Compound 9: deep red).

**Table S1:** Thermodynamic parameters (Tc, ΔTc1/2, ΔH) of DPPC bilayers in the presence of pregnane analogues 1–9 at x = 0.10 obtained from independent duplicate preparations (n = 2). Values are presented as individual replicates [Rep 1, Rep 2] and Mean plus/minus SD.

| **Compound** | **Tc (°C) [Rep 1]** | **Tc (°C) [Rep 2]** | **Tc (°C) [Mean ± SD]** | **ΔTc1/2 (°C) [Rep 1]** | **ΔTc1/2 (°C) [Rep 2]** | **ΔTc1/2 (°C) [Mean ± SD]** | **ΔH (cal/g) [Rep 1]** | **ΔH (cal/g) [Rep 2]** | **ΔH (cal/g) [Mean ± SD]** | **ΔH (kJ/mol) [Mean ± SD]** |
|---|---|---|---|---|---|---|---|---|---|---|
| **1** | 41.1 | 40.9 | 41.0 ± 0.14 | 2.4 | 2.2 | 2.3 ± 0.14 | 10.8 | 10.2 | 10.5 ± 0.42 | 32.2 ± 1.3 |
| **2** | 38.5 | 38.3 | 38.4 ± 0.14 | 2.9 | 2.7 | 2.8 ± 0.14 | 10.7 | 10.1 | 10.4 ± 0.42 | 31.9 ± 1.3 |
| **3** | 39.2 | 39.0 | 39.1 ± 0.14 | 4.1 | 3.9 | 4.0 ± 0.14 | 10.5 | 9.9 | 10.2 ± 0.42 | 31.3 ± 1.3 |
| **4** | 40.7 | 40.5 | 40.6 ± 0.14 | 2.0 | 1.8 | 1.9 ± 0.14 | 9.3 | 8.7 | 9.0 ± 0.42 | 27.6 ± 1.3 |
| **5** | 39.9 | 39.7 | 39.8 ± 0.14 | 2.9 | 2.7 | 2.8 ± 0.14 | 10.6 | 10.0 | 10.3 ± 0.42 | 31.6 ± 1.3 |
| **6** | 38.3 | 38.1 | 38.2 ± 0.14 | 2.9 | 2.7 | 2.8 ± 0.14 | 12.1 | 11.3 | 11.7 ± 0.57 | 35.9 ± 1.7 |
| **7** | 42.1 | 41.9 | 42.0 ± 0.14 | 1.7 | 1.5 | 1.6 ± 0.14 | 12.3 | 11.5 | 11.9 ± 0.57 | 36.5 ± 1.7 |
| **8** | 39.7 | 39.5 | 39.6 ± 0.14 | 1.7 | 1.5 | 1.6 ± 0.14 | 8.7 | 8.1 | 8.4 ± 0.42 | 25.8 ± 1.3 |
| **9** | 38.7 | 38.5 | 38.6 ± 0.14 | 1.7 | 1.5 | 1.6 ± 0.14 | 9.2 | 8.6 | 8.9 ± 0.42 | 27.3 ± 1.3 |
| **DPPC** | 41.3 | 41.1 | 41.2 ± 0.14 | 1.3 | 1.1 | 1.2 ± 0.14 | 10.4 | 9.8 | 10.1 ± 0.42 | 31.0 ± 1.3 |

Note: Enthalpy values in kJ/mol were calculated using 1 cal = 4.184 J and Mw(DPPC) = 734.04 g/mol. For independent duplicate preparations (n = 2), sample standard deviations were calculated based on the difference between Replicate 1 and Replicate 2 divided by the square root of two. The observed deviation of ±0.14 °C corresponds to an experimental difference of 0.20 °C between runs, consistent with the instrumental reproducibility limit of the DSC-7 system.
